# Extra-osseous talotarsal stabilization and sinus tarsi impingement syndrome: case report

**DOI:** 10.1186/1757-1146-6-S1-P6

**Published:** 2013-05-31

**Authors:** Jeffery L Jenkins

**Affiliations:** 1 School of Science and Health, University of Western Sydney, Campbelltown, NSW, 2560, Australia; 2 The Institute of Surgical Podiatry, Sydney, NSW, 2000, Australia; 3 Nepean Podiatry, Penrith, NSW, 2750, Australia

## 

Pain at the anterior-lateral aspect of the ankle is common in people with flat feet. Sinus tarsi impingement syndrome (STIS), with osseous impingement between the talus and calcaneus, is often attributed to this pain. Impingement occurs with talotarsal subluxation and reduced sinus tarsi aperture commonly associated with excessive pronation.

Level 3 evidence supports the surgical insertion of extra-osseous talotarsal stabilization (EOTTS) devices to realign and stabilize the normal range of movement at the rearfoot. A case report of a 33-year-old female diagnosed with left STIS, and successfully treated with the insertion of an EOTTS is outlined here.

A 2 year history of left ankle pain was reported to be associated with increasing intensity and duration of activity. Pain at its worst was rated as 8/10. A Foot Posture Index score of 9 and radiographs revealed a subluxed talus. Local anaesthetic injection into the sinus tarsi provided pain relief. Previous treatment with orthoses had varied success, however the patient requested a more permanent solution.

Postoperatively, follow-up of the case patient was positive without complication. Consistent with current literature, improvements were found in pain management with complete relief of symptoms reported, reductions in excessive subtalar joint pronation and Foot Posture Index (4).

EOTTS devices represent a viable minimally invasive permanent treatment option for STIS. Referral for a surgical consult is an option when conservative management of STIS is not consistently effective and/or patients request a more permanent solution.

